# LC-MS metabolomics comparisons of cancer cell and macrophage responses to methotrexate and polymer-encapsulated methotrexate

**DOI:** 10.1016/j.ijpx.2019.100036

**Published:** 2019-11-12

**Authors:** Mohammad Ahmad Al-Natour, Ali Alazzo, Amir M. Ghaemmaghami, Dong-Hyun Kim, Cameron Alexander

**Affiliations:** a School of Pharmacy, University of Nottingham, University Park, Nottingham NG72RD, UK; b Department of Pharmacy, Faculty of Pharmacy & Medical Sciences, University of Petra, Amman, Jordan; c College of Pharmacy, University of Mosul, Mosul, Iraq; d School of Life Sciences, University of Nottingham, University Park, Nottingham NG72RD, UK

**Keywords:** Polymer nanoparticles, Metabolomics, Drug delivery, Cancer nanotechnology, Methotrexate

## Abstract

Methotrexate (MTX) is a folate analogue antimetabolite widely used for the treatment of rheumatoid arthritis and cancer. A number of studies have shown that MTX delivered via nanoparticle carriers is more potent against cancer cells than free MTX, a phenomenon attributed to higher cellular uptake of the particles compared to the saturable folate receptor pathway. In this study, a cell-based global metabolic profiling approach was applied to study the effects of MTX in both free drug form and when encapsulated in -poly(lactide-co-glycolide) (PLGA) nanoparticles on a cancer cell line, A549, and also on human-like THP-1 macrophages. The results showed that MTX loaded nanoparticles had less impact on the macrophages than free MTX, and the effects on macrophages were limited to changes in nucleotide metabolism and suppression of the tricarboxylic acid cycle, whereas free MTX also led to a drop in glycolytic activity and impairment in redox homeostasis. In contrast, MTX loaded nanoparticles showed a greater impact on A549 cells than the free drug, which was in accord with studies in other cell lines in prior literature with MTX-carrier nanoparticles.

## Graphical abstract

**Figure:**
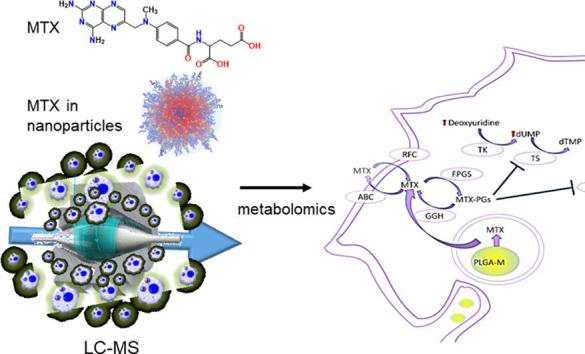

## 1 Introduction

The folate antagonist methotrexate (MTX), is used widely as an antiproliferative agent in cancer treatments and as an anti-inflammatory, particularly for rheumatoid arthritis. The mechanism of action is not completely understood for MTX yet, despite the drug first being introduced ~70 years ago as a cancer treatment (Wojtuszkiewicz et al., 2015). MTX is known to inhibit the enzyme dihydrofolate reductase (DHFR) irreversibly (Lin and Gerson, 2014), thus blocking the catalytic conversion of dihydrofolate to the active form tetrahydrofolate (THF). In turn, THF is essential for de novo purine, pyrimidine and neocluside thymidine synthesis which are required for DNA, RNA and thymidylates synthesis, MTX stops cell proliferation in the S phase, during which DNA replication takes place, by inhibiting nucleic acid and protein synthesis (Quéméneur et al., 2003). High-dose MTX has been used to treat malignancies (e.g., acute lymphoblastic leukaemia (Lonnerholm et al., 2009), non-Hodgkin lymphoma (Canellos et al., 1981), osteosarcoma (van Dalen and de Camargo, 2009), and colon cancer (Singh et al., 2006)), and to manage the symptoms of autoimmune diseases such as rheumatoid arthritis (RA) (Mikkelsen et al., 2011) at a lower dose. RA is a systemic chronic inflammatory joint disease, which is characterized by persistent synovitis, where macrophages are abundant and play a pivotal role (Wang et al., 2012). Inhibition of DHFR is not enough to manage RA; therefore, MTX is assumed to have other mechanisms including inhibition of purine metabolism by inhibiting certain enzymes that are involved in that process. These include selective suppression of B cells, preventing T cell activation, downregulation of methyltransferase activity and inhibiting interleukin 1-beta binding to its receptor on the cell surface (Böhm, 2004, Brody et al., 1993, Wessels et al., 2008).

Malignant cells are highly proliferative, and DNA replication is essential for their survival, therefore these cells express high de novo nucleotide synthesis (Tong et al., 2009). MTX is capable of stopping the growth of cancer cells by inhibiting nucleobase synthesis, however, this effect of MTX can be counteracted by administration of folic acid. In contrast, although macrophages are not proliferative cells, the effects of MTX on macrophages are not modified by folic acid. These findings suggest that the mechanism of action of MTX is not limited to the inhibition of DNA replication and is cell-line dependent.

The mechanism by which MTX enters cells affects its activity, kinetics and fate. MTX is structurally similar to folic acid, it binds to folate receptors and is known to enter cells through active carrier-mediated transport (reduced folate carrier RFC) which makes it readily available in the cytoplasm (Evans et al., 1986, Matherly and Hou, 2008). Once MTX is free in the cytosol, folylpolyglutamate synthase (FPGS) catalyses conversion of MTX to MTX-polyglutamate, rendering MTX no longer subject to the folate efflux pathway and prolonging retention inside the cells. In addition, polyglutamation increases the affinity of MTX for thymidylate synthetase (TS), phosphoribosyl aminoimidazole carboxamide formyltransferase and phosphoribosyl glycinamide formyltransferase (GART) (McBride et al., 2012, Wessels et al., 2008). The loading of MTX in nanoparticle carriers has been reported to enhance its activity in cell culture assays (Gulfam et al., 2017, Maleki et al., 2017), which may be related to a change in the mechanism by which MTX enters cells when encapsulated. This is because many nanoparticles enter cells by passive endocytosis, which is not a saturable process, unlike the active transport of free MTX entry by reduced folate receptors (Matherly and Hou, 2008).

Metabolomics methods potentially allow all the end products of every cellular process to be measured, and any alterations in metabolite levels might act as signals which can describe the effects of certain stimuli on cells very comprehensively (Nicholson and Lindon, 2008, Tiziani et al., 2011). We therefore chose LC-MS based metabolite profiling to investigate the effects of free MTX and MTX loaded NPs on selected cancer cells and macrophages. The resultant global metabolic profile data provide an explanation for the differences in cellular processing between fast proliferative cancer cells and non-proliferative human-like macrophages when challenged with MTX in free drug form and when encapsulated in polymeric nanoparticles.

## 2 Materials and methods

### 2.1 Materials

RPMI 1640 medium, heat-inactivated FBS, penicillin, streptomycin, l-glutamine, Phorbol 12-myristate 13-acetate (PMA) and formaldehyde were purchased from Sigma-Aldrich. Ammonium carbonate was purchased from Fluka. Isopropanol and acetonitrile were LC-MS grade and dichloromethane, methanol, diethyl ether and acetone were HPLC grade provided by Fisher Scientific. Fisher Scientific provided Trypan blue 0.4% and all HPLC/LC-MS grade solvents.

THP-1 (human monocyte from acute monocytic leukaemia, ATCC® TIB-202™) and A549 (human adenocarcinoma alveolar basal epithelial, ATCC® CCL-185™) cells were cultured in RPMI and DMEM media respectively, supplemented with 10% FBS, 2 mM l-glutamine, 100 μg/mL streptomycin and 100 U/mL penicillin, in a humidified atmosphere containing 5% CO2. The cells were used at passage number between 5 and 15.

### 2.2 Nanoparticles preparation and charechterisztion

Nanoparticles were prepared using Fluorescein amine labelled PLGA to follow the cellular uptake. The fluorescently labelled PLGA was synthesised by using a fluorophore with an active nucleophilic moiety (Fluoresceinamine, isomer I) as initiator in a solvent-free ring opening polymerisation (ROP) reaction of lactide (LA) and glycolide (GA) monomers according to a previously described method (Al-Natour, 2019). Nanoparticles of these fluorescein amine labelled PLGA polymers were fabricated by a solvent precipitation from DMSO into aqueous suspension followed by dialysis in a procedure modified from a previous method (Gulfam et al., 2017). Accordingly, a sample (25 mg) of the PLGA polymer, dissolved in 10 mL DMSO, was introduced into Milli-Q water (10 mL) under vigorous stirring, using a syringe pump with a flow rate of 0.70 mL min^−1^. The solution was stirred for 10 min at room temperature and NPs were purified by dialysis overnight against 1 L of Milli-Q water using a cellulose dialysis membrane (Spectrapor, cut-off 3500) to remove the DMSO. These NPs contained only fluorescent label and no drug and hence were termed ‘blank NPs’. Drug loaded NPs were prepared by a similar procedure, in which 2 mg MTX and 25 mg polymer were dissolved in 10 mL DMSO. Following precipitation in Milli-Q water and dialysis using the same procedure as for the blank NPs, the polymer NP suspensions were filtered through a membrane syringe filter (pore size: 0.22 μm) (Millex-LG, Millipore Co., USA) before further characterisation. Determination of drug contents and encapsulation efficiencies were performed by dissolving a known amount (5 mg) of freeze dried MTX-loaded NPs in DMSO. The quantification of MTX was evaluated using UV–Vis spectrophotometry (monitoring at λmax = 304 nm). The amount of loaded drug was calculated using a standard curve of MTX in DMSO. Drug content (wt%) and encapsulation efficiency (wt%) were calculated according to the following equations:

$$Drugcontent\left(wt\%\right)=\frac{weightofMTXinNPs}{weightofpolymerused}X100$$

$$Encapsulationefficiency\left(wt\%\right)=\frac{weightofMTXinNPs}{TotalweightofMTXused}X100$$

The in vitro drug release studies of MTX-loaded NPs were carried out in PBS, pH 7.4, whereas 5 mg of freeze dried MTX-NPs were re-dispersed in 2 mL PBS (pH 7.4) and the solution was placed in a dialysis device (Slide-A-Lyzer™ mini dialysis device, 3.5 K MWCO, Thermo Scientific). The NP suspensions were dialysed against 45 mL of release media (1 × PBS, pH 7.4) at 37 °C and samples (1 mL) were taken at appropriate time points and replaced with 1 mL fresh medium. The collected samples were freeze dried and dissolved in DMSO. The amount of MTX was calculated using UV–Vis spectrophotometry (λmax = 304 nm) via a standard calibration curve of MTX in DMSO, Table S1.

### 2.3 Metabolic activity (AlamarBlue®) assay

In 250 µL of fully supplemented medium, THP-1 and A549 cells were seeded on 48-well plates at densities of 250 × 10^3^ cell/cm^2^ and 25 × 10^3^ cell/cm^2^ respectively. After 24 h, the cells were washed with PBS and treated with Blank NPs, MTX loaded PLGA NPs, and free MTX for 24 h. Thereafter, the cells were washed with PBS and the culture media were replaced with fresh fully supplemented media containing 10% AlamarBlue® reagent for 4 h. Finally, 100 μL were taken from each well and transferred to 96 black well plates and the fluorescence was measured on a TECAN plate reader at excitation/emission of 540/580 nm. Six replicates from each condition were prepared and analysed. The results are plotted as mean % viability vs control ± standard error of the mean (SEM).

### 2.4 Cellular uptake study

For confocal microscopy, cells were seeded into 8-well chamber slides (obtained from Ibidi) at a seeding density of 3 × 10^5^ cell/cm^2^ in 300 µL of fully supplemented medium. After 24 h, the cells were treated with MTX loaded PLGA NPs for 3 and 24 h. Then, the cells were washed with PBS three times, fixed with 4% paraformaldehyde for 15 min, stained with Hoechst (20 µM in PBS for 10 min) for the nucleus, stained with phalloidin Alexafluor 647 for cytoskeleton and covered with mounting medium.

### 2.5 Metabolomic analysis

#### 2.5.1 MTX treatment and metabolite extraction

THP-1 and A549 cells were seeded on T25 flasks (6 replicates for each condition) at a density of 2 × 10^5^ cell/cm^2^ and 25 × 10^3^ cell/cm^2^ fully supplemented RPMI 1640 media and DMEM media respectively containing 50 ng/ml PMA. After 24 h, the cells were treated with free MTX, MTX loaded PLGA NPs and blank PLGA NPs for 24 h. Then, after removing the medium, the cells were washed briefly once with pre-warmed PBS at 37 °C. The cellular metabolism was rapidly-quenched and the metabolites were extracted simultaneously by adding 0.5 mL of methanol (−48 °C), and cell handling after quenching was performed on ice. The cells were then scraped and transferred to precooled fresh tubes at 4 °C. Cell solution was vortexed vigorously for 1 h at 4 °C and centrifuged at 17000 × *g* for 10 min at 4 °C. After the centrifugation, the supernatant was removed and dried under vacuum at room temperature. The metabolite extract was reconstituted using 70 µL of pre-cooled methanol (4 °C). 10 µL aliquots were taken from each sample to make a pooled QC in order to assess instrument performance (Want et al., 2010).

#### 2.5.2 Liquid chromatography-mass spectrometry (LC-MS) and data processing

LC-MS analysis and data processing was performed according to a previously described procedure (Alazzo et al., 2019). Briefly, LC was performed on a ZIC-pHILIC 5 μm, 4.6 × 150 mm column from Merck Sequant (Watford, UK), using an Accela LC system with a mobile phase consisting of A: 20 mM ammonium carbonate and B: 100% acetonitrile as previously described (Creek et al., 2011, Surrati et al., 2016). Chromatographic separation was carried out using the following linear gradient: 20% A (0 min) to 95% A at 15 min to 20% A at 17 min and held to 24 min. The flow rate was 300 μL min^−1^ and the injection volume was 10 μL. Samples were maintained at 4 °C, and the column was maintained at 45 °C.

MS was performed on an Orbitrap Exactive MS (Thermo Fisher Scientific, Hemel Hempstead, UK) with ESI running in positive and negative ionisation modes. Spectra were acquired in full MS scan in the range of *m*/*z* 70–1400. The capillary and probe temperatures were maintained at 275 and 150 °C, respectively. The instrument calibration was performed by modified Thermo calibration mixture masses with inclusion of C_2_H_6_NO_2_ (*m*/*z* 76.0393) for positive ion electrospray ionisation and C_3_H_5_O_3_ (*m*/*z* 89.0244) for negative ion electrospray ionisation in order to extend the calibration mass range to small metabolites.

##### 2.5.2.1 Data analysis and metabolite identification

Raw LC-MS data from the control group (untreated cells), the treatment groups (Free MTX and MTX loaded PLGA NPs, blank unloaded NPs), and reagent blanks were acquired using Xcalibur v2.1 software (Thermo Scientific, Hemel Hempstead UK), and processed with XCMS for untargeted peak-picking (Tautenhahn et al., 2008). Peak matching and related peak annotation were performed using mzMatch (Scheltema et al., 2011) and noise filtering and putative metabolite identification were then carried out using IDEOM with the default parameters (Creek et al., 2012). Metabolites that were matched with accurate masses and retention times of authentic standards were identified with Level 1 metabolite identification according to the metabolomics standards initiative (Sumner et al., 2007, Sumner et al., 2014), but when standards were not available, metabolites were identified by employing predicted retention times considered as putative (Level 2 identification). Pooled QC samples were injected randomly in between every 5–6 samples to validate system suitability and stability (Want et al., 2010). Multivariate data analysis was employed to assess changes in the cell metabolome between the control and each treatment group using orthogonal partial least squares discriminant analysis (OPLS-DA) using SIMCA-P v13.0.2 (Umetrics, Umea, Sweden) (Boccard and Rutledge, 2013). In addition to the multivariate analysis, univariate one-way ANOVA was carried out using Metaboanalyst 3.0.38 Mass ions with false discovery rate (FDR) less than 5% and variable importance in projection scores (VIP) greater than one were selected as significantly altered metabolites. The lists of significantly altered metabolites were imported to Metaboanalyst 3.0 to visualise the affected metabolic pathways (Kanehisa et al., 2014).

## 3 Results and discussion

Initial experiments demonstrated that the prepared NPs were well tolerated by both cells lines (Figure S1, S2), as evidenced by <5% changes in overall metabolic activity evaluated with Alamar Blue assays. A global LC-MS metabolic profiling approach was employed to study the effects of MTX and nanoparticles (NPs) with entrapped MTX (Table S1) on THP-1 and A549 cells respectively. Using Orbitrap coupled LC-MS, a total of 400 and 800 different metabolites were identified in THP-1 and A549 cells respectively. These included amino acids, lipids, carbohydrates, nucleotides, cofactors and energy metabolism metabolites.

**Fig. 1:**
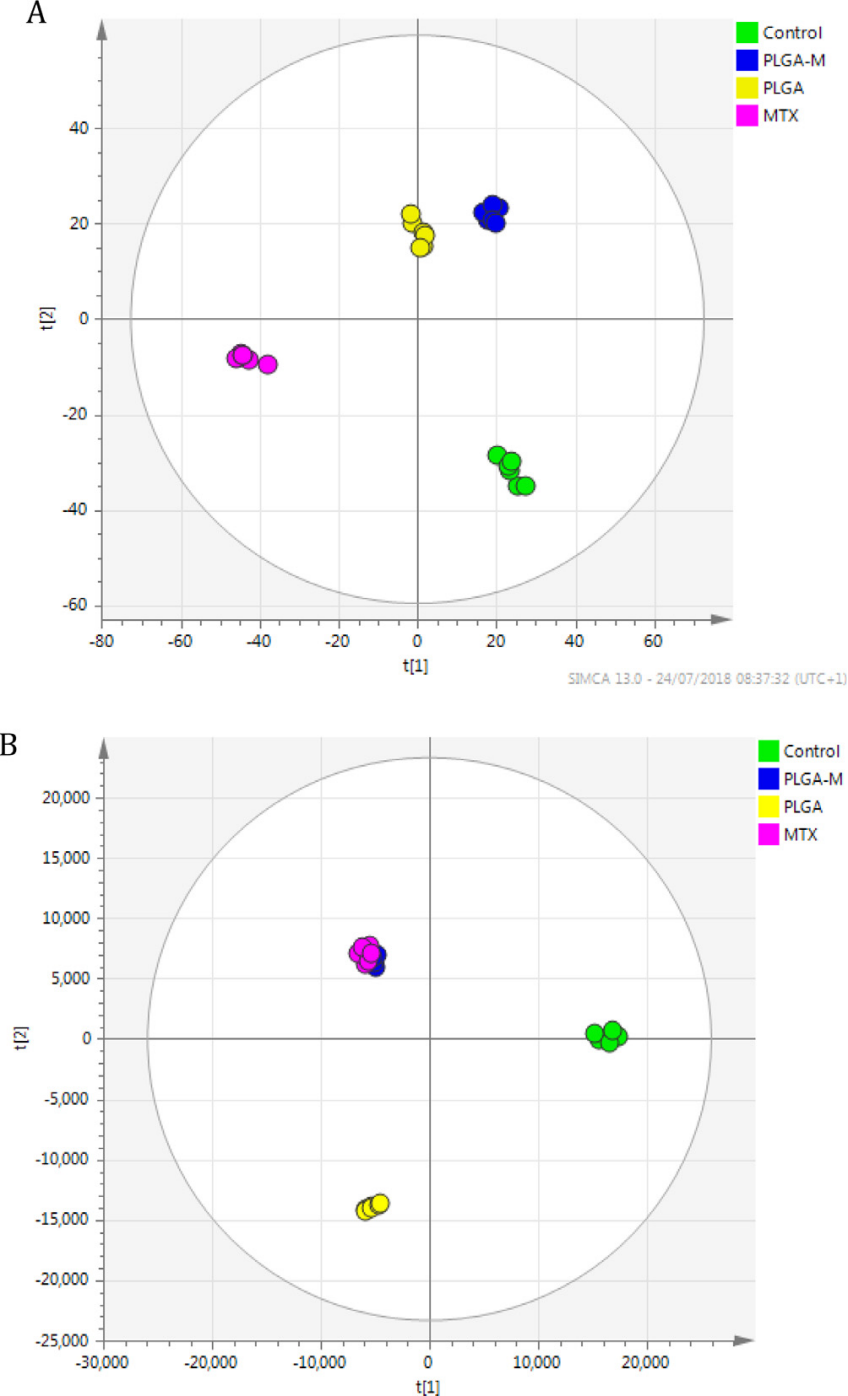
(A) OPLS-DA scores plots of THP-1 cells samples (R2X = 0.616, R2Y = 0.99 Q2 = 0.68, CV-ANOVA p-value 0.0083, n = 6). B) A549 cells samples (R2X = 0.84, R2Y = 0.98, Q2 = 0.82, CV-ANOVA p-value 1.7e-6, n = 6) after 24 h treatment with free MTX, MTX loaded PLGA NPs and blank PLGA NPs. (the control group without any treatment).

Metabolic alterations were assessed primarily by OPLS-DA where a clear separation between the tested groups was observed, and the two cell lines showed different responses to free MTX, MTX loaded NPs and unloaded ‘blank’ PLGA NPs. The OPLS-DA plot for THP-1 cells (Fig. 1A) shows that the cells were sensitive to the treatment groups with NPs more than to free MTX, whereas MTX loaded NPs and blank PLGA NPs treated groups clustered close to each other. This implies that both MTX loaded PLGA NPs and the PLGA-only (i.e. ‘blank’)NPs affected the cells in a similar manner, even though MTX is a potent drug and PLGA has been widely regarded as cytocompatible and is in existing clinical use in humans. The metabolic changes observed in these cases can thus be interpreted as a consequence of the phagocytic nature of THP-1 cells. The presence of the NPs, which were of similar dimensions and charges (100–120 nm and between −27 and −47 mV, Table S1, ESI) to viral particles, might be expected to have activated strongly any phagocytosis processes and their accompanying metabolic changes (Saborano et al., 2017) in ways that may have been analogous to those in processing exogenous small molecule components. Indeed, Saborano et al noted that macrophages exposed to a range of nanoparticles, including PLGA, expressed metabolic changes which inferred an inflammatory M1-type response. These were manifest in upregulation of glycolysis and the TCA cycle metabolites and thus were indicative of a phagocytic behavior in the presence of the NPs. However, it should be noted that the concentration of NPs in our study was 5-fold less than that used by Saborano et al, and thus while we therefore anticipated a phagocytic response to all the NPs in the study, the extent of the changes observed in macrophage metabolism in the presence of the blank NPs were in fact low compared to those induced by MTX-loaded NPs.

As expected, the proliferative A549 cells were more sensitive to MTX, where MTX treated groups were not separable (see Fig. 1B), despite the fact that one group was treated with free MTX and the other was exposed to MTX loaded PLGA NPs. This indicated that MTX loaded NPs caused similar metabolic alterations in A549 cells to those caused by free MTX.

Two-stage statistical analysis combining multivariate and univariate analysis was adopted to study further the key metabolites upon the different treatments to both cell lines. Metabolites responsible for the separation in the OPLS-DA models having VIP values greater than 1 were selected as changing metabolites. In addition, one way ANOVA was employed to enhance the statistical power of this study and the metabolites with FDR of <0.05 were further studied, Table S2, S3. Non-proliferative activated THP-1 macrophages were expected to tolerate MTX well because THP-1 cells lose their proliferative ability once activated into macrophages, and MTX exerts its effect during the S phase in cellular proliferation (Richter et al., 2016). Surprisingly, free MTX resulted in a decrease in the TCA cycle metabolites (2-oxoglutarate, citrate and *cis*-aconitate) and in glycolytic activities (phosphoenolpyruvate, 3-phospho-d-glycerate and d-gluconic acid), as shown in Table 1. Energy metabolism has a main role in macrophage polarisation, which results in either M1 macrophages that encourage inflammation, or in M2 macrophages that suppress inflammation. It is well known that M1 polarisation depends on glycolysis and M2 polarisation relies on fatty acid oxidation (FAO) (Huang et al., 2014, Pearce and Pearce, 2013). The drop in glycolysis and the absence of any signs of FAO hyperactivity (no significant decrease in crinite or increase in acylcarnitine) suggest that the macrophages were, however, not extensively polarised in these assays. In addition, downregulation of the TCA cycle results in depletion of ATP, which is essential for macrophage activation. These results suggest that impairing cellular energy production could be an important mechanism by which MTX controls the symptoms of RA by preventing macrophage polarisation to the inflammatory type M1. The pentose phosphate pathway is the main source of NADPH in the cell, and cellular redox homeostasis is maintained via NADPH which is used to recycle glutathione from its oxidised form. The results showed that administration of free MTX decreased the intracellular glutathione levels, this could be attributed to the drop in glucose metabolism, characterised by the depletion of gluconic acid. Unexpectedly, MTX loaded NPs did not affect the glycolytic activity, nor were the glutathione levels decreased. Another distinct fingerprint difference between the two types of macrophages (M1 and M2) is arginine metabolism, whereby the first type converts arginine to nitric oxide, which endows macrophages with cytostatic or cytotoxic activity against microorganisms, and the second type converts arginine to ornithine which is an anabolic promoter of cell proliferation. THP-1 cells treated with free MTX showed higher levels of ornithine, by a factor of 1.59: 1 compared to controls, potentially indicating an enhanced polarisation to an M2 anti-inflammatory macrophage phenotype, while for the MTX-containing NPs the factor was 1.05:1. Metabolic profiling results also showed that free MTX had a greater impact on the metabolism of THP-1 cells in comparison to NPs loaded with MTX. In this case, MTX suppressed glycolytic activity, disturbed redox homeostasis, decreased TCA cycle activity and altered nucleotide metabolism (see Table 1), while MTX loaded NPs changed only TCA cycle activity and nucleotide metabolism. The variations in effects on the cell lines likely arose due to the differences between the concentrations of MTX in the cytoplasm. In the case of the drug alone, MTX would have been rapidly available following internalisation, whereas the NPs loaded MTX would have been trapped in phagosomes and inaccessible during the initial stages in the cytoplasm, which is where all glycolytic activities take place. Over time, the phagosomes in macrophages fuse with lysosomes to produce phagolysosomes which are rich in hydrolytic enzymes and reactive oxygen species. MTX in free drug form is susceptible to oxidative degradation (Barisci et al., 2015), whereas MTX entrapped in a nanoparticle core would have been less accessible to oxidants.

**Table 1:** Biologically relevant metabolites which changed significantly in THP-1 and A549 cells after treatment with free MTX and MTX loaded NPs, IDC: metabolite identification level according to metabolomics standards initiative L1 – Level 1, L2 – Level 2. Colours represent changes in metabolite levels with blue indicating a decrease and red denoting an increase.

	THP-1 cells					
Mass	RT	FORMULA	Putative metabolite	IDC	MTX NPS	Free MTX
	Redox homeostasis					
307.0838	9.80	C_10_H_17_N_3_O_6_S	Glutathione	L1	1.01	0.65
612.1523	11.00	C_20_H_32_N_6_O_12_S_2_	Glutathione disulfide	L1	0.80	0.97
132.0898	16.19	C_5_H_12_N_2_O_2_	Ornithine	L1	1.46	1.91
	Energy metabolism					
146.0215	10.32	C_5_H_6_O_5_	2-Oxoglutarate	L2	0.71	0.59
192.0270	11.72	C_6_H_8_O_7_	Citrate	L2	0.70	0.43
174.0164	11.29	C_6_H_6_O_6_	*cis*-Aconitate	L2	0.71	0.39
167.9823	11.42	C_3_H_5_O_6_P	Phosphoenolpyruvate	L1	1.02	0.43
	Nucleotide Metabolism					
286.0567	10.72	C_7_H_15_N_2_O_8_P	GAR	L2	17.12	8.20
308.0411	9.41	C_9_H_13_N_2_O_8_P	dUMP	L1	1017	705
228.0747	7.84	C_9_H_12_N_2_O_5_	Deoxyuridine	L2	7.88	9.20
	A549 Cells					
Mass	RT	FORMULA	Putative metabolite		MTX NPS	Free MTX
	Redox homeostasis and apoptotic markers					
307.0837	9.77	C_10_H_17_N_3_O_6_S	Glutathione	L1	0.72	0.74
306.0759	10.83	C_20_H_32_N_6_O_12_S_2_	Glutathione disulfide	L1	0.93	0.84
183.0661	10.15	C_5_H_14_NO_4_P	Choline phosphate	L1	0.64	0.84
103.0997	10.43	C_5_H_13_NO	Choline	L2	0.47	0.59
	Energy metabolism					
260.0297	10.46	C_6_H_13_O_9_P	Fructose 6-phosphate	L1	0.66	0.64
359.2670	5.51	C_19_H_37_NO_5_	2-Hydroxylauroylcarnitine	L2	2.29	2.03
259.1784	5.72	C_13_H_25_NO_4_	Hexanoylcarnitine	L2	2.09	2.17
397.3190	5.19	C_23_H_43_NO_4_	Hexadecenoylcarnitine	L2	1.96	2.32
425.3503	5.08	C_25_H_47_NO_4_	Elaidiccarnitine	L2	1.69	1.82
387.2983	5.49	C_21_H_41_NO_5_	2-Hydroxymyristoylcarnitine	L2	1.66	1.87
371.3034	5.28	C_21_H_41_NO_4_	Tetradecanoylcarnitine	L2	1.60	1.79
	Nucleotide Metabolism					
136.0384	8.89	C_5_H_4_N_4_O	Hypoxanthine	L1	0.81	1.42
267.0967	8.22	C_10_H_13_N_5_O_4_	Adenosine	L1	0.74	1.74
135.0545	8.64	C_5_H_5_N_5_	Adenine	L1	0.79	1.10
347.0630	9.46	C_10_H_14_N_5_O_7_P	AMP	L1	0.64	0.65
286.0565	10.56	C_7_H_15_N_2_O_8_P	GAR	L2	11.46	9.78

In order to study this further, we evaluated the transport of MTX-loaded NPs in THP-1 cells via super-resolution confocal microscopy (Fig. 3). Within 3 h of incubation, the MTX-containing nanoparticles were apparent within the cell interior, as demonstrated by distinct punctate regions of the green fluorescent NPs in the cytosol. It is noteworthy that no diffuse staining of the cytoplasm was observed in these images, indicating that the polymer NPs were retained by intracellular compartments over this time period. In turn, this could also indicate that export of MTX by the normal folate-carrier type recycling pathways did not take place, as these processes have been reported to take place with periods much less than 1 h in vitro, albeit in a panel of cancer cell lines (Paulos et al., 2006). Nevertheless, given that both internalization and export kinetics of folate-receptor pathways are very rapid, the retention of the MTX-containing NPs was indicative of a different transport process for these materials compared to MTX, and thus a likely different exposure of cellular components to MTX delivered via the PLGA carrier than as a free drug. These results also support other papers which have demonstrated that nanoparticle- or polymer surfactant-mediated transport is more effective for delivering anti-cancer agents to target cells compared to that of the free drug through modification of internalization and export pathways (Batrakova et al., 2010).

**Fig. 2:**
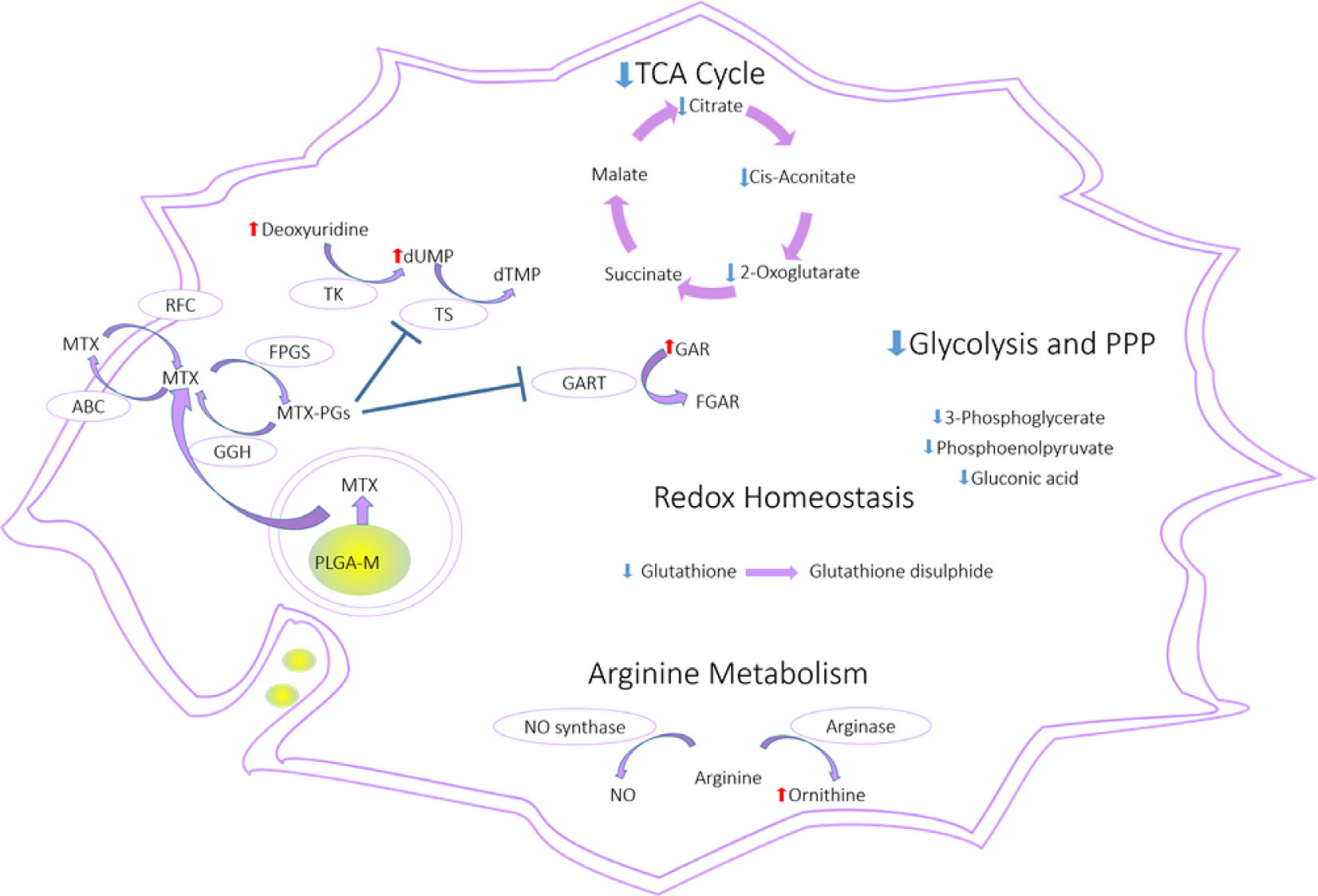
Overview of the affected pathways in the THP-1 cells after treatment with the free MTX and MTX loaded NPs. Red arrow = increased, blue arrow = decreased, Methotrexate enters the cell mainly via reduced folate receptor (RCF). Folylpolyglutamyl synthetase (FPGS) catalyses the polyglutamation of MTX to produce MTX polyglutamates (MTX-PGs). MTX-PGs are better retained intracellularly than MTX because they are not susceptible to the ATP-binding cassette (ABC) that is responsible for MTX efflux. MTX-PGs have higher affinity than MTX to thymidylate synthetase (TS) and phosphoribosylglycinamide formyltransferase (GART). Deoxyuridine-5-monophosphate (dUMP), deoxythymidine monophosphate (dTMP), 5′-phosphoribosyl-glycinamide (GAR), 5′-phosphoribosyl-N-formylglycinamide (FGAR) and nitric oxide (NO). Pathway analysis was performed using Metaboanalyst 3.0 software following LC-MS assays of cell (THP-1 and A549) extracts following incubation with MTX and MTX-loaded NPs for 24 h.

Further effects on cell processes were apparent in terms of nucleotide metabolism, which was altered both by free MTX and MTX loaded NPs. Thymidine synthesis, which starts by reduction of uridine to deoxyuridine, followed by phosphorylation to produce dUMP and methylation by thymidylate synthase to produce thymidine monophosphate was reduced. This occurred via suppression of thymidylate synthase by MTX, and as a result, the levels of deoxyuridine and dUMP were higher in MTX and MTX loaded NPs treated groups in comparison to the control groups. Secondly, de novo purine synthesis was also suppressed by both treatments, in particular, the third step where the enzyme phosphoribosylglycinamide formyltransferase (GART) catalyses the formation of 5′-phosphoribosyl-N-formylglycinamide (FGAR) from 5′-phosphoribosyl-glycinamide (GAR) resulting in accumulation of GAR, (see Fig. 2).

**Fig. 3:**
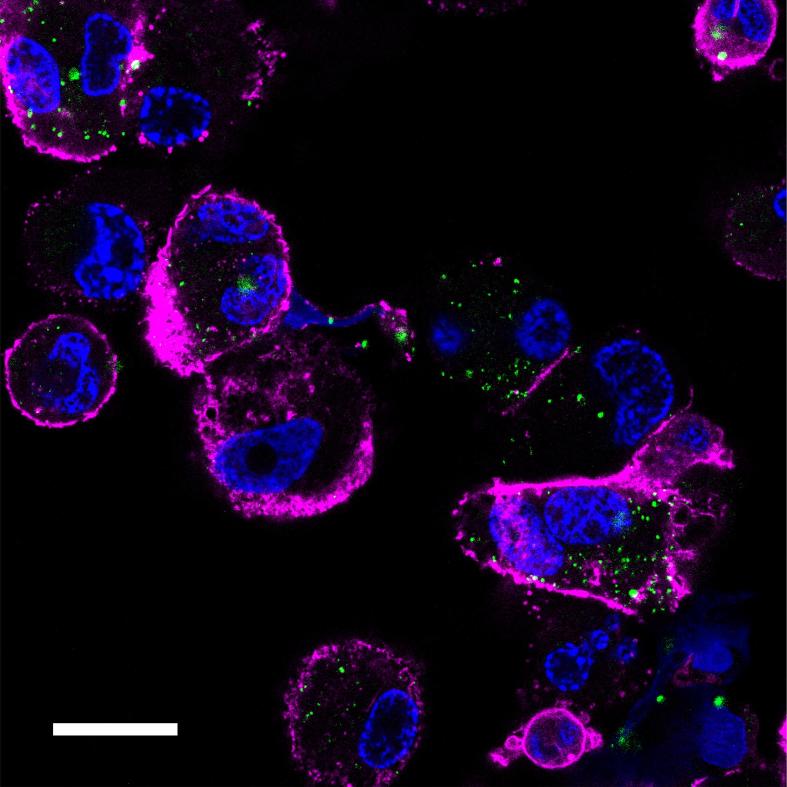
Super-resolution microscopy image of methotrexate loaded PLGA nanoparticles (green) (92 μg/mL, 3 h) in THP-1 derived macrophages. Cells were fixed and stained for cytoskeleton (Alexafluor 647, magenta) and nucleus (Hoechst nucleus, blue). Scale bar = 20 µm.

As a comparator, the A549 lung cancer cells showed early signs of apoptosis upon treatment with free MTX and MTX loaded NPs. The levels of choline, phosphocholine, and glutathione were lower compared to the untreated groups. Similar changes have been reported in apoptotic cells (Halama et al., 2013; Rainaldi et al., 2008), accompanied by increased fatty acid oxidation (FAO). The levels of several acylcarnitines i.e. hexadecanoyl-carnitine, O-hexanoyl-carnitine, palmitoyl-carnitine and 2-hydroxylauroylcarnitine, were higher in the MTX treated groups (see Table 1). A shift toward FAO can occur during energy crisis, when cells cannot produce enough ATP from glucose metabolism, or to survive oxidative stress (Carracedo et al., 2013). Cancer cells generally prefer glucose metabolism to harness energy rapidly, in a process known as the Warburg Effect (Vander Heiden et al., 2009). but also recycle glutathione from its oxidised form in order to retain cellular redox homeostasis. Free MTX and MTX loaded NPs treated groups showed a decrease in glutathione, suggestive of oxidative stress mechanisms. Indeed MTX has previously been reported to exert cytotoxic effects by inducing oxidative stress (Akacha et al., 2018) and this could explain the upregulation of FAO observed. In contrast to the effects in THP-1 cells, treating the cancer cells with MTX-loaded NPs induced more metabolic changes than when free MTX was administered. A549 cells treated with MTX-loaded NPs showed depletion in hypoxanthine, adenine, adenosine and AMP, which are four consecutive metabolites in the purine metabolism pathway. In addition, an increased GAR level indicated suppression in purine metabolism. On the other hand, free MTX resulted in depletion of AMP only, and an increase in GAR. The degree of purine synthesis inhibition could thus be considered as a measure for the potency of MTX inside the cells. The increased activity of the MTX-loaded NPs could, as noted earlier, be attributed to the fact that the NPs entered the cells via passive endocytosis, allowing higher uptake compared to the RFC-mediated transport of MTX alone (Gulfam et al., 2017). The saturation of folate-receptor pathways via MTX is well-known, whereas the internalisation routes for nanoparticles are highly cell-, and polymer-type, dependent (Zhang et al., 2015). In our case, the effects of MTX, and MTX-loaded NPs were different in the two cell lines, and thus were indicative of a modification of the cellular activities of MTX as a consequence of the delivery, rather than of the drug itself. Nevertheless, the fact that LC-MS metabolomics data were able to establish the differences in MTX activity are promising, and suggest that such an approach might be used to probe further the individual pathways along putative internalization routes. Experiments to evaluate in greater detail this hypothesis are under way and will be reported in a future manuscript.

## 4 Conclusion

In this study, THP-1 derived macrophages and A549 lung carcinoma cells were exposed to subtoxic concentrations of free MTX and MTX loaded NPs, and global metabolic profiling was performed. The results showed that free MTX had higher impacts on the metabolome than MTX-loaded NPs in THP-1 derived macrophages. In these cells, MTX induced oxidative stress, a drop in glycolytic activity, reduction in the TCA cycle, and inhibition of nucleotide metabolism. In contrast, the effects of MTX-loaded NPs were limited to alteration of nucleotide metabolism and inhibition of TCA cycle. In comparison, A549 cancer cells were more susceptible to MTX-loaded NPs than to the free drug, in terms of purine synthesis suppression, which we suggest was linked to the different entry mechanisms for nanoparticles compared to free MTX, and lack of efflux pumps for the NPs. We believe the presented work shows that metabolomics is a valuable analytical technique that can be used to understand mechanisms of action of drugs and formulations in various clinically important cell lines.

## Declaration of Competing Interest

The authors declare that they have no known competing financial interests or personal relationships that could have appeared to influence the work reported in this paper.
