# Supplementary material for: LC-MS metabolomics comparisons of cancer cell and macrophage responses to methotrexate and polymer-encapsulated methotrexate
Source: Int J Pharm X. 2019 Nov 12;1:100036. doi: 10.1016/j.ijpx.2019.100036 (PMC6977166; doi:10.1016/j.ijpx.2019.100036)
Supplement: Supplementary data 1 [file mmc1.docx]

Table S1: Size and zeta potential of the blank and MTX loaded NPs.

| **Type of NPs** | **Mean Diameter (nm)** | **Zeta potential (mv)** | |
| --- | --- | --- | --- |
| Blank NPs | 120 ± 1.4 | | -46.7 ± 2.5 |
| MTX NPs | 129 ± 2.0 | | -27.1 ± 3.3 |

Figure S1: Viability of activated THP-1 macrophages following exposure to free and polymer encapculated methotrexate for 24 hrs.

Figure S2: Viability of A549 cells following exposure to free and polymer encapculated methotrexate for 24 hrs.

Table S1: The metabolites that were changed significantly in THP-1 derived macrophages after the treatment with free and polymer-encapsulated methotrexate, IDC: metabolite identification level according to the metabolomics standards initiative L1 – Level 1, L2 – Level 2. Colours in the table represent changes in metabolite levels with blue indicating a decrease and red denoting an increase.

| **Mass** | **Polarity** | **Mass error (ppm)** | **RT** | **FORMULA** | **Putative metabolite** | **IDC** | **Map** | **Control** | **MTX** | **MTX NPS** |
| --- | --- | --- | --- | --- | --- | --- | --- | --- | --- | --- |
| 84.0211 | + | -0.22 | 8.96 | C4H4O2 | 3-Butynoate | L2 | Carbohydrate Metabolism | 1.00 | 0.42 | 0.42 |
| 109.0198 | + | 0.467 | 11.13 | C2H7NO2S | Hypotaurine | L2 | Amino Acid Metabolism | 1.00 | 0.67 | 0.86 |
| 113.0478 | - | 0.802 | 10.16 | C5H7NO2 | Pyrroline-5-carboxylate | L2 | Amino Acid Metabolism | 1.00 | 0.87 | 0.55 |
| 117.0538 | + | -0.56 | 11.71 | C3H7N3O2 | Guanidinoacetate | L1 | Amino Acid Metabolism | 1.00 | 0.95 | 0.98 |
| 117.079 | + | -0.32 | 9.25 | C5H11NO2 | Betaine | L1 | Amino Acid Metabolism | 1.00 | 0.76 | 0.84 |
| 118.0267 | - | 0.806 | 10.12 | C4H6O4 | Succinate | L1 | Carbohydrate Metabolism | 1.00 | 1.27 | 1.43 |
| 120.0423 | - | 0.303 | 10.95 | C4H8O4 | Erythrulose | L2 | Carbohydrate Metabolism | 1.00 | 1.07 | 0.93 |
| 125.0589 | + | -0.37 | 11.71 | C5H7N3O | 5-Methylcytosine | L2 | Nucleotide Metabolism | 1.00 | 1.05 | 1.13 |
| 127.0634 | + | 0.241 | 10.21 | C6H9NO2 | 2,3,4,5-Tetrahydropyridine-2-carboxylate | L2 | Amino Acid Metabolism | 1.00 | 0.67 | 0.81 |
| 129.0427 | - | 0.731 | 6.35 | C5H7NO3 | 1-Pyrroline-4-hydroxy-2-carboxylate | L2 | Amino Acid Metabolism | 1.00 | 0.99 | 0.65 |
| 130.0631 | - | 0.884 | 4.79 | C6H10O3 | 3-Methyl-2-oxopentanoic acid | L2 | Amino Acid Metabolism | 1.00 | 0.74 | 0.68 |
| 131.0583 | + | 0.655 | 10.89 | C5H9NO3 | Glutamate 5-semialdehyde | L2 | Amino Acid Metabolism | 1.00 | 0.87 | 0.83 |
| 131.0946 | + | -0.44 | 9.31 | C6H13NO2 | Leucine | L1 | Amino Acid Metabolism | 1.00 | 0.77 | 0.81 |
| 132.0534 | + | -0.75 | 8.85 | C4H8N2O3 | N-Carbamoylsarcosine | L2 | Amino Acid Metabolism | 1.00 | 1.58 | 1.26 |
| 132.0899 | + | -0.26 | 16.12 | C5H12N2O2 | Ornithine | L1 | Amino Acid Metabolism | 1.00 | 1.59 | 1.05 |
| 136.0384 | + | -0.93 | 8.98 | C5H4N4O | Hypoxanthine | L1 | Nucleotide Metabolism | 1.00 | 2.65 | 0.62 |
| 140.0585 | + | -0.61 | 7.39 | C6H8N2O2 | Methylimidazoleacetic acid | L2 | Amino Acid Metabolism | 1.00 | 0.87 | 0.76 |
| 141.0191 | - | 0.373 | 10.88 | C2H8NO4P | Ethanolamine phosphate | L1 | Amino Acid Metabolism | 1.00 | 0.95 | 1.25 |
| 145.0851 | + | -0.45 | 11.32 | C5H11N3O2 | 4-Guanidinobutanoate | L2 | Amino Acid Metabolism | 1.00 | 0.81 | 1.00 |
| 146.0691 | + | -0.67 | 10.94 | C5H10N2O3 | Glutamine | L1 | Amino Acid Metabolism | 1.00 | 0.95 | 0.94 |
| 148.0372 | - | 0.589 | 10.1 | C5H8O5 | 2-Hydroxyglutarate | L1 | Amino Acid Metabolism | 1.00 | 0.61 | 0.82 |
| 149.051 | + | -0.8 | 9.43 | C5H11NO2S | Methionine | L1 | Amino Acid Metabolism | 1.00 | 0.82 | 0.86 |
| 150.0527 | - | -0.28 | 11.04 | C5H10O5 | Xylulose | L2 | Carbohydrate Metabolism | 1.00 | 0.98 | 0.94 |
| 155.0695 | + | -0.23 | 11.18 | C6H9N3O2 | Histidine | L2 | Amino Acid Metabolism | 1.00 | 0.84 | 0.85 |
| 158.0439 | - | -0.12 | 10.86 | C4H6N4O3 | Allantoin | L1 | Nucleotide Metabolism | 1.00 | 0.87 | 0.85 |
| 159.126 | + | 0.076 | 5.65 | C8H17NO2 | FA amino(8:0) | L2 | Lipids: Fatty Acyls | 1.00 | 1.54 | 1.29 |
| 161.0687 | + | -0.65 | 9.04 | C6H11NO4 | O-Acetyl-L-homoserine | L2 | Amino Acid Metabolism | 1.00 | 0.46 | 0.73 |
| 163.0481 | - | 0.456 | 8.67 | C5H9NO5 | L-erythro-4-Hydroxyglutamate | L2 | Amino Acid Metabolism | 0.00 | 0.00 | 11.79 |
| 165.079 | + | -0.29 | 8.64 | C9H11NO2 | Phenylalanine | L2 | Amino Acid Metabolism | 1.00 | 0.83 | 0.78 |
| 169.0044 | - | 0.063 | 10.91 | C3H7NO5S | L-Cysteate | L1 | Amino Acid Metabolism | 1.00 | 0.73 | 0.82 |
| 169.9981 | - | 0.934 | 11.03 | C3H7O6P | Glyceraldehyde 3-phosphate | L2 | Carbohydrate Metabolism | 1.00 | 1.25 | 0.92 |
| 174.0791 | + | -1.34 | 7.25 | C10H10N2O | Indole-3-acetamide | L2 | Amino Acid Metabolism | 1.00 | 0.32 | 0.52 |
| 175.0633 | + | -0.47 | 4.49 | C10H9NO2 | 5-Hydroxyindoleacetaldehyde | L2 | Amino Acid Metabolism | 1.00 | 0.90 | 0.58 |
| 175.0957 | + | -0.53 | 11.4 | C6H13N3O3 | Citrulline | L1 | Amino Acid Metabolism | 1.00 | 0.88 | 0.94 |
| 179.0582 | - | 0.535 | 5.42 | C9H9NO3 | Hippurate | L1 | Amino Acid Metabolism | 1.00 | 1.04 | 0.72 |
| 181.074 | + | 0.473 | 10.21 | C9H11NO3 | Tyrosine | L1 | Amino Acid Metabolism | 1.00 | 0.88 | 0.82 |
| 182.0791 | - | 0.852 | 10.47 | C6H14O6 | Sorbitol | L2 | Carbohydrate Metabolism | 1.00 | 0.97 | 1.00 |
| 188.1273 | + | -0.33 | 18.52 | C7H16N4O2 | Homoarginine | L2 | Amino Acid Metabolism | 1.00 | 1.19 | 0.93 |
| 189.1 | - | 0.228 | 5.43 | C8H15NO4 | 2 -(Butylamido)-4-hydroxybutanoic acid | L2 | - | 1.00 | 1.11 | 81.74 |
| 203.1157 | + | -0.56 | 8.95 | C9H17NO4 | O-Acetylcarnitine | L1 | Amino Acid Metabolism | 1.00 | 0.40 | 0.44 |
| 204.1475 | + | 0.049 | 14.22 | C9H20N2O3 | 3-Hydroxy-N6,N6,N6-trimethyl-L-lysine | L2 | Amino Acid Metabolism | 1.00 | 0.82 | 0.78 |
| 208.0848 | + | -0.5 | 9.22 | C10H12N2O3 | Kynurenine | L1 | Amino Acid Metabolism | 1.00 | 0.86 | 0.81 |
| 215.0559 | - | 0.649 | 11.08 | C5H14NO6P | sn-glycero-3-Phosphoethanolamine | L2 | Lipid Metabolism | 1.00 | 0.70 | 0.81 |
| 216.0401 | - | 1.701 | 12.08 | C5H13O7P | 2-C-Methyl-D-erythritol 4-phosphate | L2 | Lipid Metabolism | 1.00 | 0.74 | 0.92 |
| 226.1066 | + | -0.42 | 11.26 | C9H14N4O3 | Carnosine | L2 | Amino Acid Metabolism | 1.00 | 0.71 | 0.76 |
| 231.1471 | + | -0.47 | 7.92 | C11H21NO4 | O-Butanoylcarnitine | L1 | Lipids: Fatty Acyls | 1.00 | 0.55 | 0.69 |
| 257.103 | + | 0.06 | 10.51 | C8H20NO6P | sn-glycero-3-Phosphocholine | L1 | Lipid Metabolism | 1.00 | 0.87 | 0.79 |
| 259.1784 | + | -0.56 | 7.29 | C13H25NO4 | O-hexanoyl-R-carnitine | L2 | Lipids: Fatty Acyls | 1.00 | 0.47 | 0.45 |
| 264.1045 | + | -0.4 | 18.46 | C12H16N4OS | Thiamin | L2 | Metabolism of Cofactors and Vitamins | 1.00 | 0.92 | 0.79 |
| 278.1519 | - | 0.989 | 4.26 | C16H22O4 | Alpha-CEHC | L2 | - | 1.00 | 0.77 | 0.86 |
| 299.2824 | + | -0.67 | 6.21 | C18H37NO2 | FA (16:2) | L2 | Lipids: Fatty Acyls | 1.00 | 0.94 | 1.19 |
| 324.0361 | - | 1.312 | 10.05 | C9H13N2O9P | UMP | L1 | Nucleotide Metabolism | 1.00 | 1.07 | 1.11 |
| 369.2879 | + | -0.46 | 5.39 | C21H39NO4 | cis-5-Tetradecenoylcarnitine | L2 | - | 1.00 | 0.48 | 0.49 |
| 371.3036 | + | -0.48 | 5.36 | C21H41NO4 | Tetradecanoylcarnitine | L2 | - | 1.00 | 0.40 | 0.43 |
| 384.1217 | + | -0.2 | 10.15 | C14H20N6O5S | Adenosyl-L-homocysteine | L2 | Amino Acid Metabolism | 1.00 | 1.16 | 1.29 |
| 425.3505 | + | -0.37 | 5.14 | C25H47NO4 | Elaidiccarnitine | L2 | - | 1.00 | 0.49 | 0.49 |
| 427.3662 | + | -0.32 | 5.11 | C25H49NO4 | Stearoylcarnitine | L2 | - | 1.00 | 0.51 | 0.61 |
| 446.0604 | - | 0.915 | 10.9 | C11H20N4O11P2 | CDP-ethanolamine | L2 | Lipid Metabolism | 1.00 | 1.00 | 1.41 |
| 472.1126 | + | 0.182 | 9.85 | C14H26N4O10P2 | CMP-N-trimethyl-2-aminoethylphosphonate | L2 | Lipid Metabolism | 1.00 | 2.50 | 2.19 |
| 479.3011 | + | -0.39 | 5.04 | C23H46NO7P | PE (18:1) | L2 | Lipids: Glycerophospholipids | 1.00 | 1.30 | 1.11 |
| 488.1077 | + | 0.513 | 10.32 | C14H26N4O11P2 | CDP-choline | L2 | Lipid Metabolism | 1.00 | 1.88 | 1.70 |
| 523.291 | - | 1.192 | 4.39 | C24H46NO9P | PS (18:1) | L2 | Lipids: Glycerophospholipids | 1.00 | 1.41 | 1.06 |
| 593.575 | - | 1.884 | 4.57 | C38H75NO3 | SP (20:0) | L2 | Lipids: Sphingolipids | 1.00 | 1.87 | 1.46 |
| 619.59 | + | -0.32 | 4.53 | C40H77NO3 | Cer(d-40:2) | L2 | Lipids: Sphingolipids | 1.00 | 1.26 | 1.04 |
| 673.5045 | + | 0.019 | 4.56 | C37H72NO7P | PE(P-32:1) | L2 | Lipids: Glycerophospholipids | 1.00 | 0.77 | 0.98 |
| 729.5309 | + | 0.136 | 4.73 | C40H76NO8P | PC(32:2) | L2 | Lipids: Glycerophospholipids | 1.00 | 0.78 | 1.00 |
| 733.5628 | + | 0.978 | 4.72 | C40H80NO8P | PC(32:0) | L2 | Lipids: Glycerophospholipids | 1.00 | 0.93 | 1.19 |
| 759.5774 | + | -0.58 | 4.7 | C42H82NO8P | PC(34:1) | L2 | Lipids: Glycerophospholipids | 1.00 | 0.85 | 1.03 |
| 776.5571 | - | 1.592 | 4.22 | C42H81O10P | PG(36:1) | L2 | Lipids: Glycerophospholipids | 1.00 | 0.70 | 1.18 |
| 787.609 | + | -0.23 | 4.7 | C44H86NO8P | PC(36:1) | L2 | Lipids: Glycerophospholipids | 1.00 | 0.85 | 0.99 |
| 820.5258 | - | 1.093 | 4.07 | C46H77O10P | PG(40:7) | L2 | Lipids: Glycerophospholipids | 1.00 | 0.78 | 0.83 |
| 879.5838 | + | -0.06 | 4.17 | C45H86NO13P | PI (36:0) | L2 | Lipids: Glycerophospholipids | 1.00 | 1.02 | 0.87 |
| 883.535 | + | -1.65 | 4.07 | C50H78NO10P | PS(44:10) | L2 | Lipids: Glycerophospholipids | 1.00 | 0.90 | 1.00 |

Table S1: The metabolites that were changed significantly in A549 cells after the treatment with free and polymer-encapsulated methotrexate, IDC: metabolite identification level according to the metabolomics standards initiative L1 – Level 1, L2 – Level 2. Colours in the table represent changes in metabolite levels with blue indicating a decrease and red denoting an increase.

| **Mass** | **Polarity** | **Mass error (ppm)** | **RT** | **FORMULA** | **Putative metabolite** | **IDC** | **Map** | **Control** | **MTX** | **MTX NPs** |
| --- | --- | --- | --- | --- | --- | --- | --- | --- | --- | --- |
| 78.0139 | + | -1.518 | 7.25 | C2H6OS | Mercaptoethanol | L2 | Medium Component | 1 | 4.44 | 3.78 |
| 84.0211 | + | -1.519 | 10.36 | C4H4O2 | 3-Butynoate | L2 | Carbohydrate Metabolism | 1 | 1.45 | 1.44 |
| 89.0477 | + | -1.619 | 11.19 | C3H7NO2 | Alanine | L1 | Amino Acid Metabolism | 1 | 1.06 | 1.03 |
| 103.0633 | + | -2.095 | 11.18 | C4H9NO2 | 4-Aminobutanoate | L2 | Amino Acid Metabolism | 1 | 0.68 | 0.62 |
| 105.0426 | + | -1.48 | 6.03 | C3H7NO3 | 2-Amino-3-hydroxypropanoic acid | L2 | Lipids: Fatty Acyls | 1 | 1.16 | 1.03 |
| 105.079 | + | -1.534 | 15.28 | C4H11NO2 | Diethanolamine | L2 | Lipid Metabolism | 1 | 0.27 | 0.19 |
| 109.0198 | + | -1.41 | 11.04 | C2H7NO2S | Hypotaurine | L2 | Amino Acid Metabolism | 1 | 0.9 | 0.97 |
| 112.1252 | + | -1.744 | 4.5 | C8H16 | 3-methyl-1-heptene | L2 | Lipids: Fatty Acyls | 1 | 1.29 | 1.08 |
| 113.0477 | + | -1.325 | 7.26 | C5H7NO2 | (S)-1-Pyrroline-5-carboxylate | L2 | Amino Acid Metabolism | 1 | 1.48 | 1.57 |
| 113.0589 | + | -1.32 | 8.59 | C4H7N3O | Creatinine | L2 | Amino Acid Metabolism | 1 | 1.14 | 1.11 |
| 117.0538 | + | -1.97 | 11.64 | C3H7N3O2 | Guanidinoacetate | L1 | Amino Acid Metabolism | 1 | 1.13 | 1.03 |
| 117.079 | + | -1.62 | 9.12 | C5H11NO2 | Betaine | L1 | Amino Acid Metabolism | 1 | 1.39 | 1.35 |
| 122.048 | + | -1.42 | 7.32 | C6H6N2O | Nicotinamide | L1 | Metabolism of Cofactors and Vitamins | 1 | 1.28 | 1.09 |
| 127.0382 | + | -1.162 | 9.12 | C4H5N3O2 | 5-Amino-4-imidazole carboxylate | L2 | Nucleotide Metabolism | 0 | 94.12 | 103.21 |
| 129.0426 | + | -1.274 | 5.9 | C5H7NO3 | 1-Pyrroline-3-hydroxy-5-carboxylate | L2 | Amino Acid Metabolism | 1 | 1.34 | 0.98 |
| 129.0426 | + | -1.326 | 7.46 | C5H7NO3 | 1-Pyrroline-4-hydroxy-2-carboxylate | L2 | Amino Acid Metabolism | 1 | 1.14 | 0.97 |
| 129.079 | + | -1.539 | 7.22 | C6H11NO2 | N4-Acetylaminobutanal | L2 | Amino Acid Metabolism | 1 | 0.79 | 0.77 |
| 131.0583 | + | -1.449 | 10.75 | C5H9NO3 | L-Glutamate 5-semialdehyde | L2 | Amino Acid Metabolism | 1 | 0.71 | 0.69 |
| 131.0583 | + | -1.424 | 5.57 | C5H9NO3 | N-Acetyl-beta-alanine | L2 | Amino Acid Metabolism | 1 | 1.12 | 1.23 |
| 131.0695 | + | -1.542 | 10.94 | C4H9N3O2 | Creatine | L1 | Amino Acid Metabolism | 1 | 1.01 | 0.9 |
| 132.0899 | + | -1.856 | 16.19 | C5H12N2O2 | L-Ornithine | L1 | Amino Acid Metabolism | 1 | 1.46 | 1.28 |
| 136.0636 | + | -1.929 | 20.58 | C7H8N2O | 1-Methylnicotinamide | L2 | Metabolism of Cofactors and Vitamins | 1 | 0.88 | 1.02 |
| 140.0586 | + | -1.851 | 5.68 | C6H8N2O2 | Methylimidazoleacetic acid | L2 | Amino Acid Metabolism | 1 | 0.41 | 0.36 |
| 144.0422 | + | -1.786 | 10.37 | C6H8O4 | 2,3-Dimethylmaleate | L2 | Metabolism of Cofactors and Vitamins | 1 | 1.35 | 1.46 |
| 145.0739 | + | -1.792 | 5.64 | C6H11NO3 | 4-Acetamidobutanoate | L2 | Amino Acid Metabolism | 1 | 0.44 | 0.37 |
| 145.0851 | + | -2.031 | 11.25 | C5H11N3O2 | 4-Guanidinobutanoate | L2 | Amino Acid Metabolism | 1 | 1.02 | 0.92 |
| 145.1103 | + | -1.784 | 10 | C7H15NO2 | 4-Trimethylammoniobutanoate | L1 | Amino Acid Metabolism | 1 | 1.25 | 1.2 |
| 146.0691 | + | -2.163 | 10.85 | C5H10N2O3 | Glutamine | L1 | Amino Acid Metabolism | 1 | 2.06 | 2.22 |
| 146.1054 | + | -2.359 | 17.25 | C6H14N2O2 | Lysine | L2 | Amino Acid Metabolism | 1 | 1.39 | 1.25 |
| 147.0531 | + | -1.988 | 9.99 | C5H9NO4 | Glutamate | L2 | Amino Acid Metabolism | 1 | 1.77 | 1.54 |
| 161.0688 | + | -1.865 | 8.95 | C6H11NO4 | O-Acetyl-L-homoserine | L2 | Amino Acid Metabolism | 1 | 0.27 | 0.21 |
| 169.0504 | + | -2.021 | 9.73 | C4H12NO4P | Phosphodimethylethanolamine | L2 | Lipid Metabolism | 1 | 0.79 | 0.71 |
| 169.0739 | + | -1.696 | 7.48 | C8H11NO3 | Pyridoxine | L2 | Metabolism of Cofactors and Vitamins | 1 | 1.58 | 1.43 |
| 174.1117 | + | -1.571 | 18.34 | C6H14N4O2 | Arginine | L1 | Amino Acid Metabolism | 1 | 1.41 | 1.21 |
| 175.0634 | + | -1.673 | 4.37 | C10H9NO2 | 5-Hydroxyindoleacetaldehyde | L2 | Amino Acid Metabolism | 1 | 1.74 | 1.66 |
| 179.0795 | + | -1.268 | 10.39 | C6H13NO5 | Glucosamine | L2 | Carbohydrate Metabolism | 1 | 1.46 | 1.49 |
| 181.074 | + | -1.151 | 10.19 | C9H11NO3 | Tyrosine | L1 | Amino Acid Metabolism | 1 | 1.38 | 1.24 |
| 182.0788 | - | -2.875 | 10.44 | C6H14O6 | Sorbitol | L2 | Carbohydrate Metabolism | 1 | 1.16 | 1.22 |
| 183.0661 | + | -1.574 | 10.15 | C5H14NO4P | Choline phosphate | L1 | Lipid Metabolism | 1 | 0.84 | 0.64 |
| 188.1525 | + | -1.732 | 15.8 | C9H20N2O2 | N6,N6,N6-Trimethyl-L-lysine | L2 | Amino Acid Metabolism | 1 | 1.02 | 0.87 |
| 193.0741 | + | -0.91 | 5.04 | C10H11NO3 | Phenylacetylglycine | L2 | Amino Acid Metabolism | 1 | 1.36 | 1.31 |
| 203.1157 | + | -1.933 | 8.85 | C9H17NO4 | O-Acetylcarnitine | L1 | Amino Acid Metabolism | 1 | 0.68 | 0.72 |
| 204.0899 | + | -1.895 | 9.68 | C11H12N2O2 | Tryptophan | L1 | Amino Acid Metabolism | 1 | 0.86 | 0.78 |
| 211.0357 | + | -2.163 | 10 | C4H10N3O5P | Phosphocreatine | L2 | Amino Acid Metabolism | 1 | 0.89 | 0.68 |
| 217.1313 | + | -2.242 | 8.28 | C10H19NO4 | O-Propanoylcarnitine | L2 | Lipids: Fatty Acyls | 1 | 0.72 | 0.6 |
| 219.1107 | + | -1.906 | 7.22 | C9H17NO5 | Pantothenate | L1 | Amino Acid Metabolism | 1 | 1.4 | 1.29 |
| 257.1028 | + | -2.086 | 10.43 | C8H20NO6P | sn-glycero-3-Phosphocholine | L1 | Lipid Metabolism | 1 | 0.66 | 0.5 |
| 262.0888 | + | -2.115 | 7.43 | C12H14N4OS | Thiamine aldehyde | L2 | Metabolism of Cofactors and Vitamins | 1 | 2.17 | 2.11 |
| 264.1044 | + | -2.209 | 18.82 | C12H16N4OS | Thiamin | L2 | Metabolism of Cofactors and Vitamins | 1 | 0.86 | 0.98 |
| 279.2562 | + | -1.95 | 4.86 | C18H33NO | Linoleamide | L2 | Lipids: Fatty Acyls | 1 | 1.33 | 1.21 |
| 297.0895 | + | -2.202 | 7.24 | C11H15N5O3S | 5'-Methylthioadenosine | L1 | Amino Acid Metabolism | 1 | 1.24 | 1.14 |
| 304.2401 | - | -2.723 | 4.12 | C20H32O2 | FA (20:4) | L2 | Lipids: Fatty Acyls | 1 | 2.26 | 1.07 |
| 306.2557 | - | -2.8 | 4.12 | C20H34O2 | Icosatrienoic acid | L2 | Lipids: Fatty Acyls | 1 | 1.42 | 0.72 |
| 307.0837 | + | -2.296 | 9.77 | C10H17N3O6S | Glutathione | L2 | Amino Acid Metabolism | 1 | 0.74 | 0.72 |
| 371.3034 | + | -2.105 | 5.28 | C21H41NO4 | Tetradecanoylcarnitine | L2 | - | 1 | 1.79 | 1.6 |
| 397.319 | + | -1.963 | 5.19 | C23H43NO4 | Hexadec-2-enoylcarnitine | L2 | - | 1 | 2.32 | 1.96 |
| 399.3347 | + | -2.01 | 5.15 | C23H45NO4 | O-Palmitoyl-R-carnitine | L2 | Lipids: Fatty Acyls | 1 | 2.14 | 1.84 |
| 425.3503 | + | -1.936 | 5.08 | C25H47NO4 | Elaidiccarnitine | L2 | - | 1 | 1.82 | 1.69 |
| 437.2903 | - | -2.645 | 4.99 | C21H44NO6P | PE (16:1) | L2 | Lipids: Glycerophospholipids | 1 | 1.57 | 1.24 |
| 467.301 | + | -1.636 | 5.31 | C22H46NO7P | PC (14:0) | L2 | Lipids: Glycerophospholipids | 1 | 2.07 | 1.57 |
| 479.301 | + | -1.707 | 4.97 | C23H46NO7P | PE (18:1) | L2 | Lipids: Glycerophospholipids | 1 | 1.7 | 1.32 |
| 481.3531 | + | -1.439 | 5.23 | C24H52NO6P | PC (16:2) | L2 | Lipids: Glycerophospholipids | 1 | 1.55 | 1.1 |
| 493.3165 | + | -1.818 | 5.23 | C24H48NO7P | PC (16:1) | L2 | Lipids: Glycerophospholipids | 1 | 2.13 | 1.51 |
| 495.3322 | + | -1.689 | 5.18 | C24H50NO7P | PC (16:0) | L2 | Lipids: Glycerophospholipids | 1 | 2.05 | 1.48 |
| 507.3687 | + | -1.439 | 5.13 | C26H54NO6P | PC (18:1) | L2 | Lipids: Glycerophospholipids | 1 | 1.55 | 1.09 |
| 519.3328 | + | -0.408 | 5.15 | C26H50NO7P | PC (18:2) | L2 | Lipids: Glycerophospholipids | 1 | 2.95 | 2.17 |
| 537.5117 | - | -2.498 | 4.5 | C34H67NO3 | SP (16:0) | L2 | Lipids: Sphingolipids | 1 | 1.47 | 1.54 |
| 721.5042 | + | -1.725 | 4.43 | C41H72NO7P | PE(P-36:4) | L2 | Lipids: Glycerophospholipids | 1 | 1.3 | 1.29 |
| 723.5199 | + | -1.616 | 4.44 | C41H74NO7P | PE(P-36:4) | L2 | Lipids: Glycerophospholipids | 1 | 1.36 | 1.29 |
| 743.5464 | + | -1.349 | 4.51 | C41H78NO8P | PE(36:2) | L2 | Lipids: Glycerophospholipids | 1 | 1.46 | 1.26 |
| 747.5199 | + | -1.714 | 4.42 | C43H74NO7P | PE (38:7) | L2 | Lipids: Glycerophospholipids | 1 | 1.3 | 1.25 |
| 765.5308 | + | -1.376 | 4.43 | C43H76NO8P | PE(38:5) | L2 | Lipids: Glycerophospholipids | 1 | 1.32 | 1.36 |
| 767.5464 | + | -1.525 | 4.44 | C43H78NO8P | PE(38:4) | L2 | Lipids: Glycerophospholipids | 1 | 1.33 | 1.34 |
| 771.6139 | + | -1.712 | 4.66 | C44H86NO7P | PC (36:1) | L2 | Lipids: Glycerophospholipids | 1 | 1.24 | 0.98 |
| 787.5359 | - | -2.214 | 4.09 | C42H78NO10P | PS(36:2) | L2 | Lipids: Glycerophospholipids | 1 | 0.96 | 0.9 |
| 787.6085 | + | -2.266 | 4.73 | C44H86NO8P | PC(36:1) | L2 | Lipids: Glycerophospholipids | 1 | 1.29 | 1.09 |
| 807.5776 | + | -1.793 | 4.57 | C46H82NO8P | PC(38:5) | L2 | Lipids: Glycerophospholipids | 1 | 1.38 | 1.27 |
| 809.5936 | + | -1.318 | 4.58 | C46H84NO8P | PC(38:4) | L2 | Lipids: Glycerophospholipids | 1 | 1.33 | 1.18 |
| 883.536 | + | -2.269 | 3.95 | C50H78NO10P | PS(44:10) | L2 | Lipids: Glycerophospholipids | 1 | 1.32 | 1.19 |
